# Supplementary figures and images for: NASP functions in the cytoplasm to prevent histone H3 aggregation during early embryogenesis
Source: J Cell Biol. 2026 May 6;225(7):e202511182. doi: 10.1083/jcb.202511182 (PMC13148224; doi:10.1083/jcb.202511182)

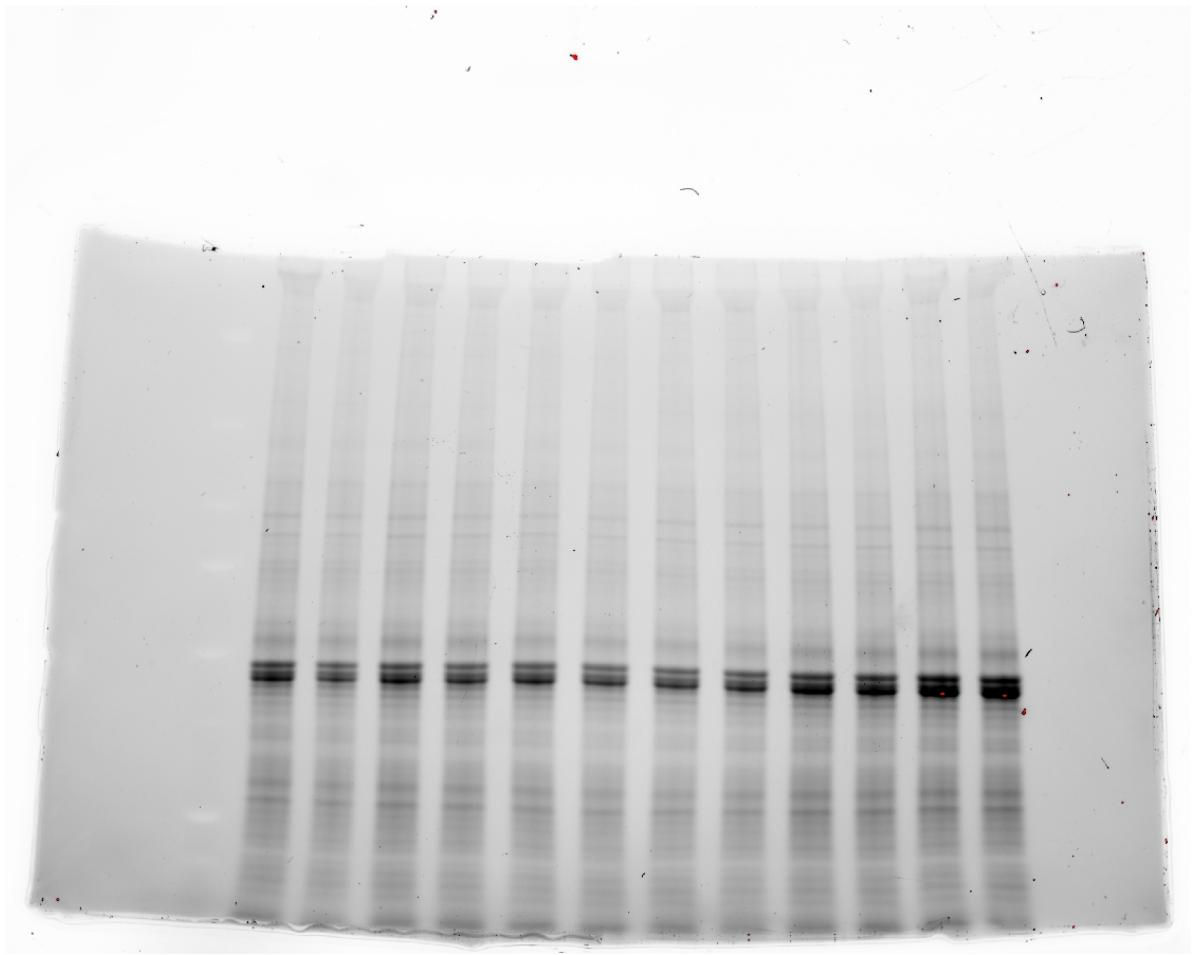

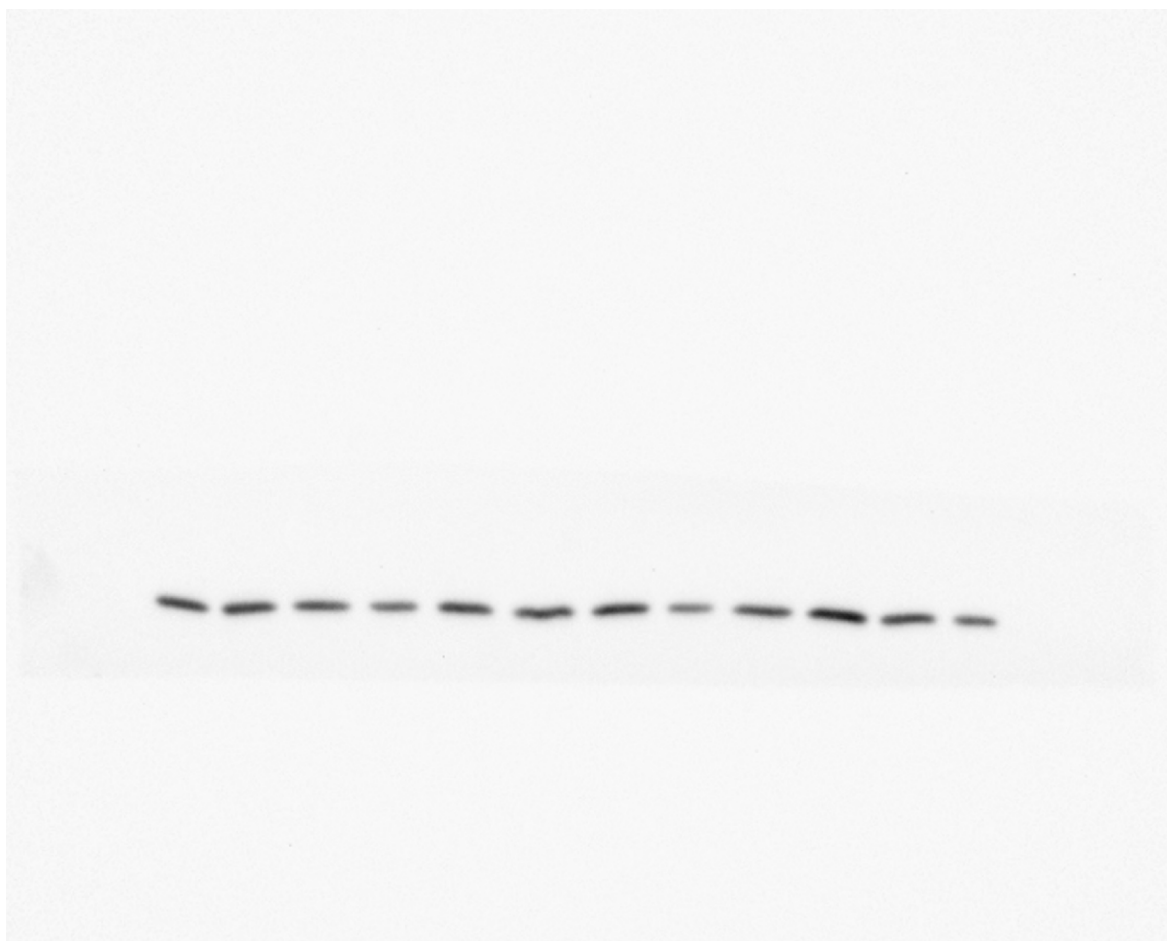

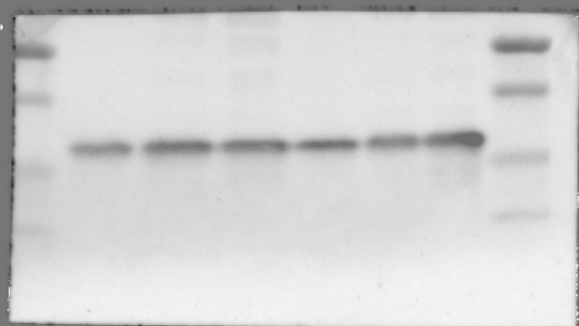

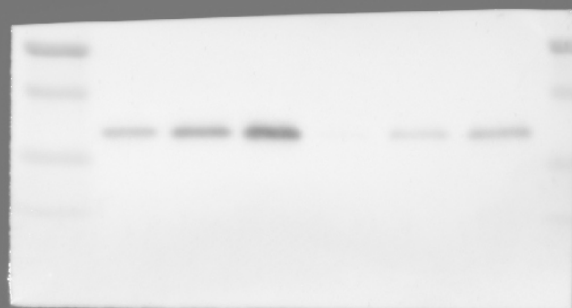

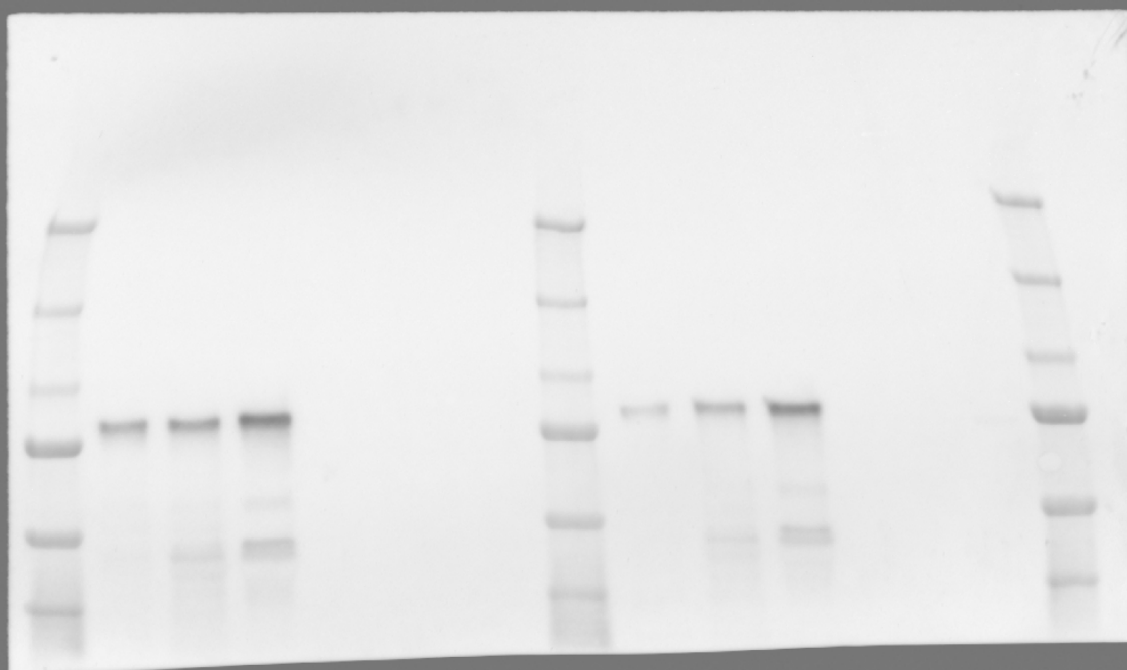

Supplement: SourceData F4 — is the source file for Fig. 4. [file jcb_202511182_sourcedataf4.pdf]

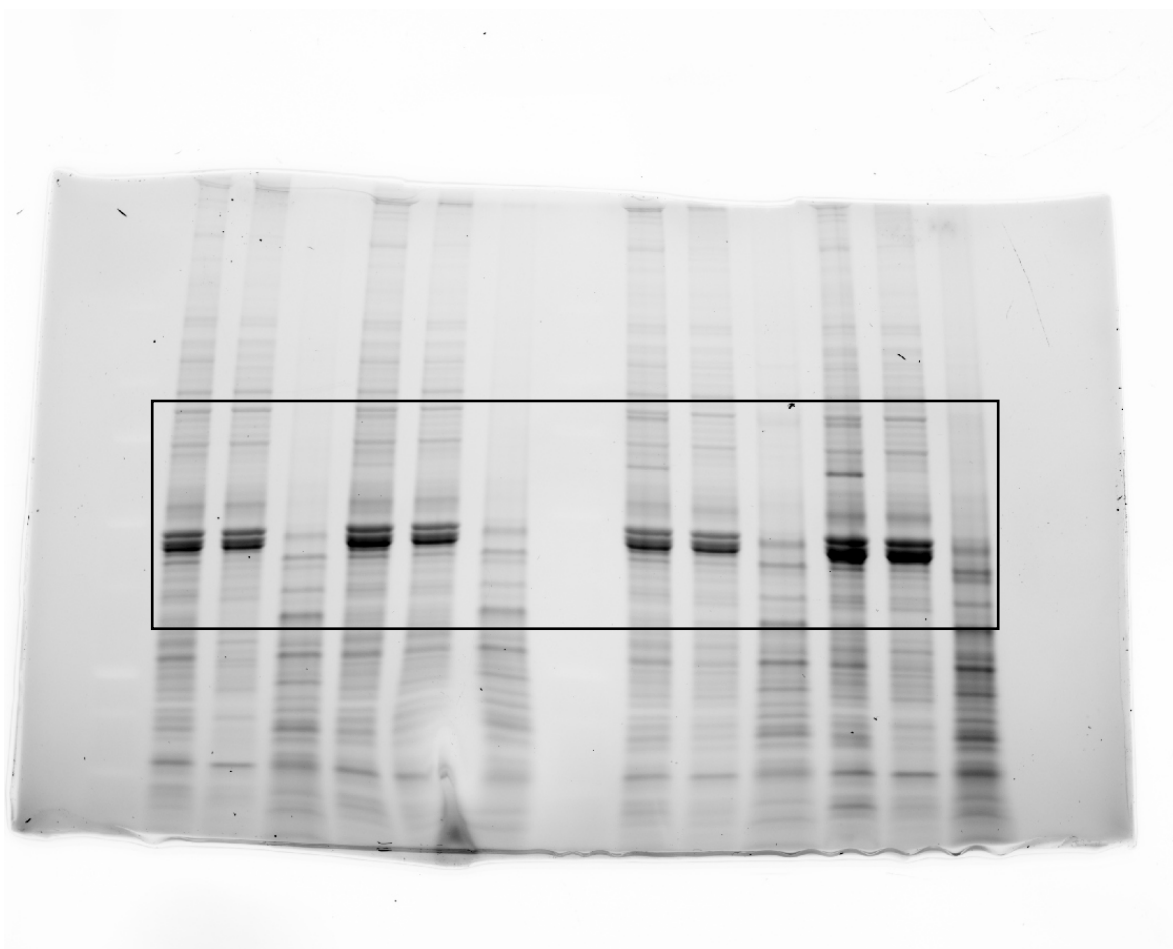

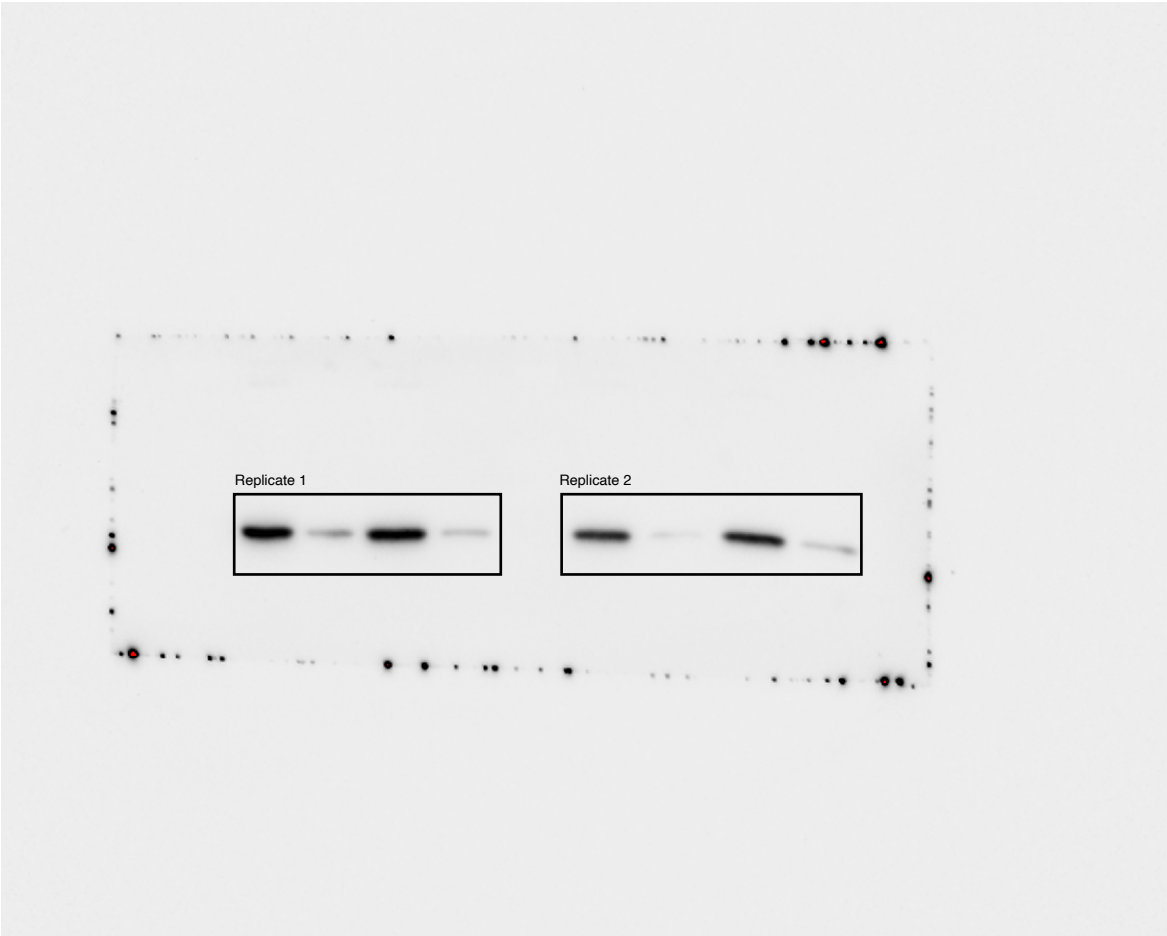

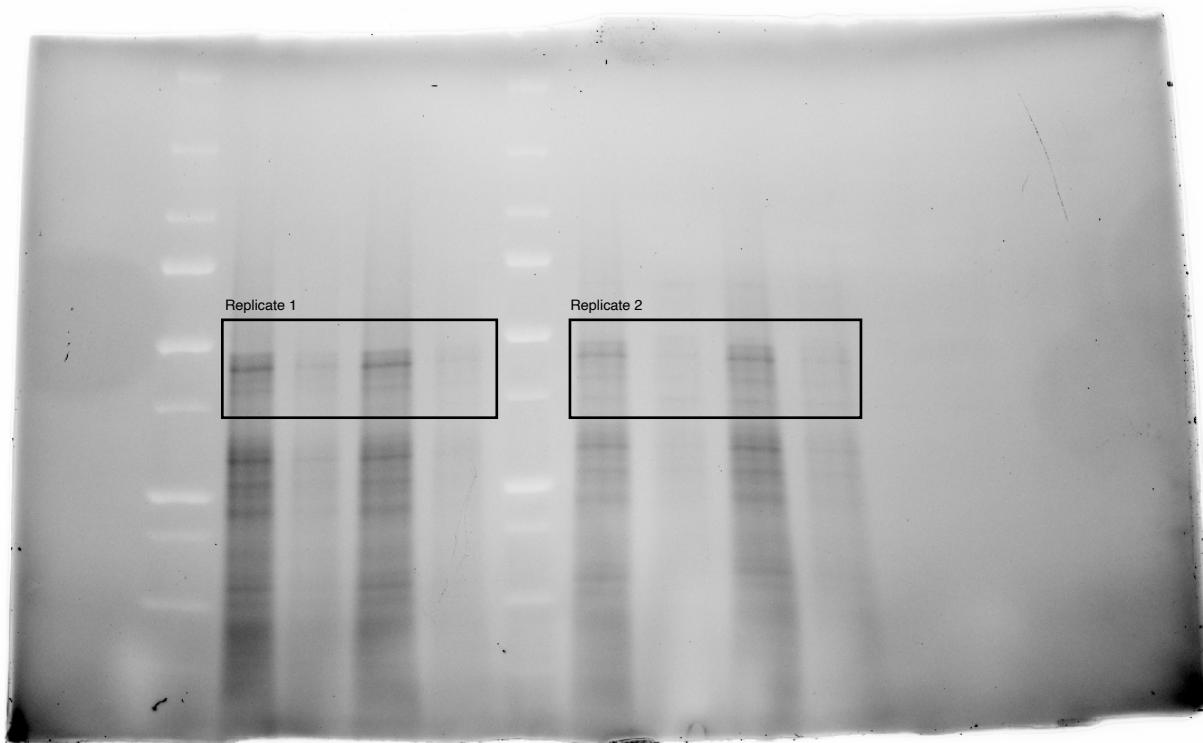

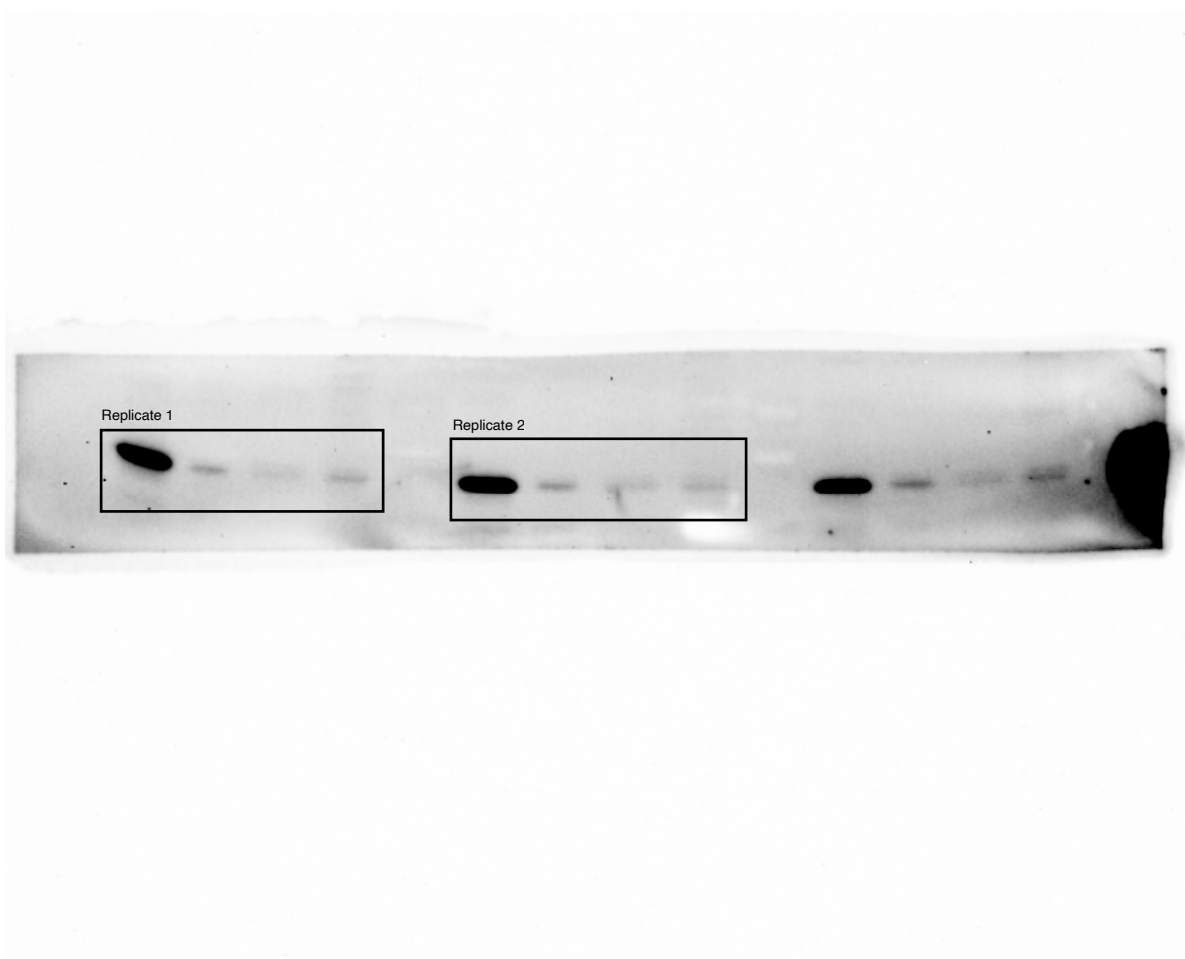

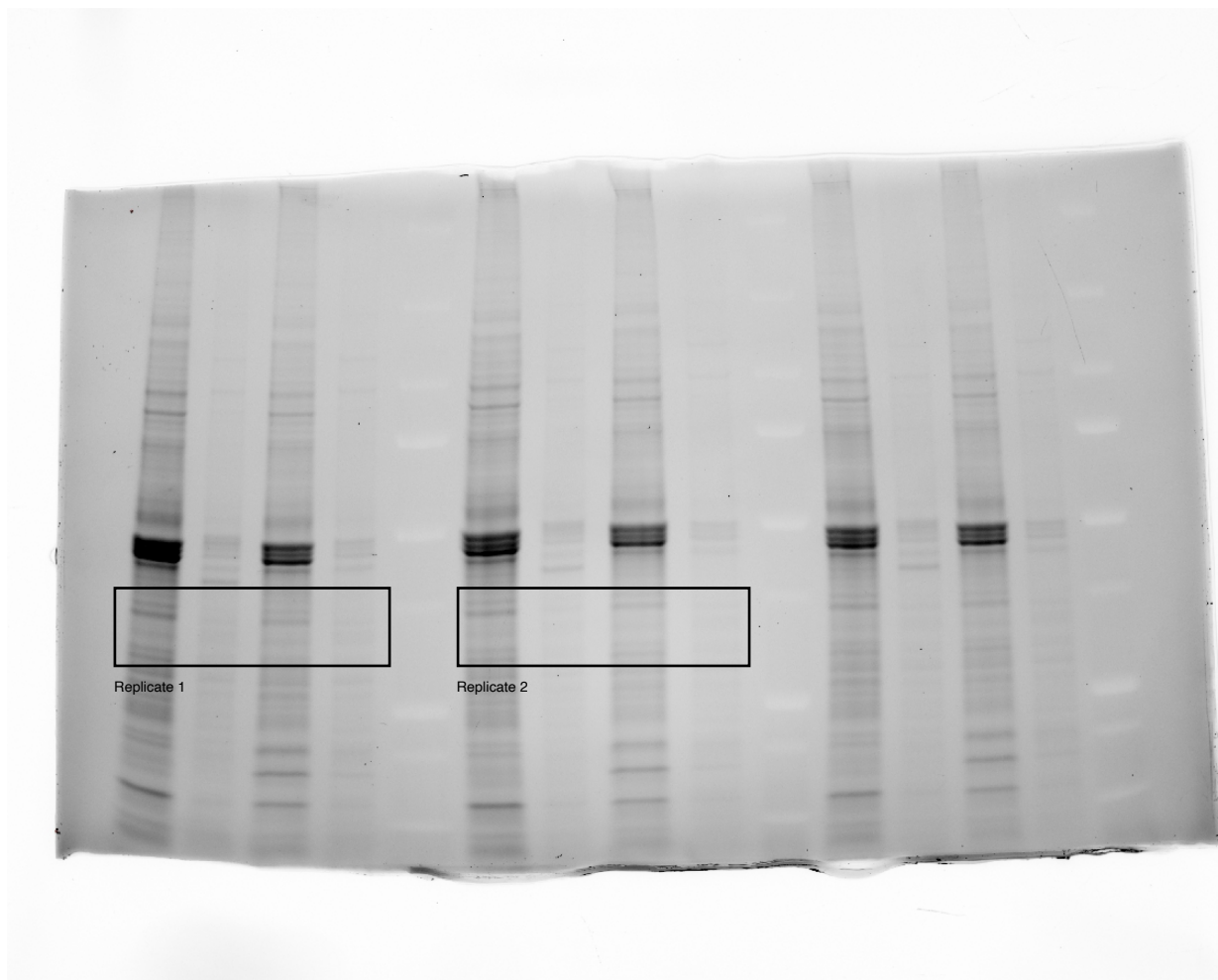

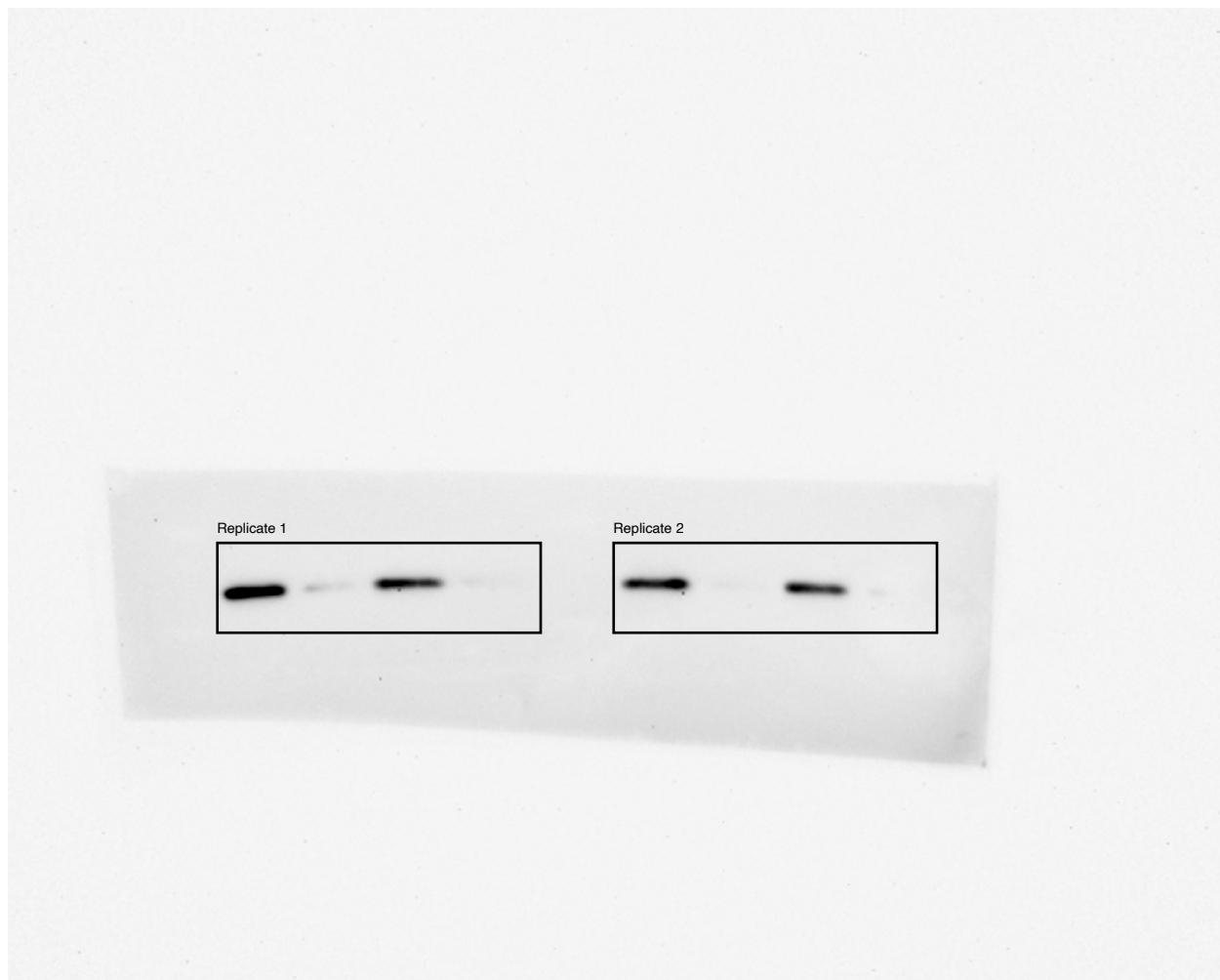

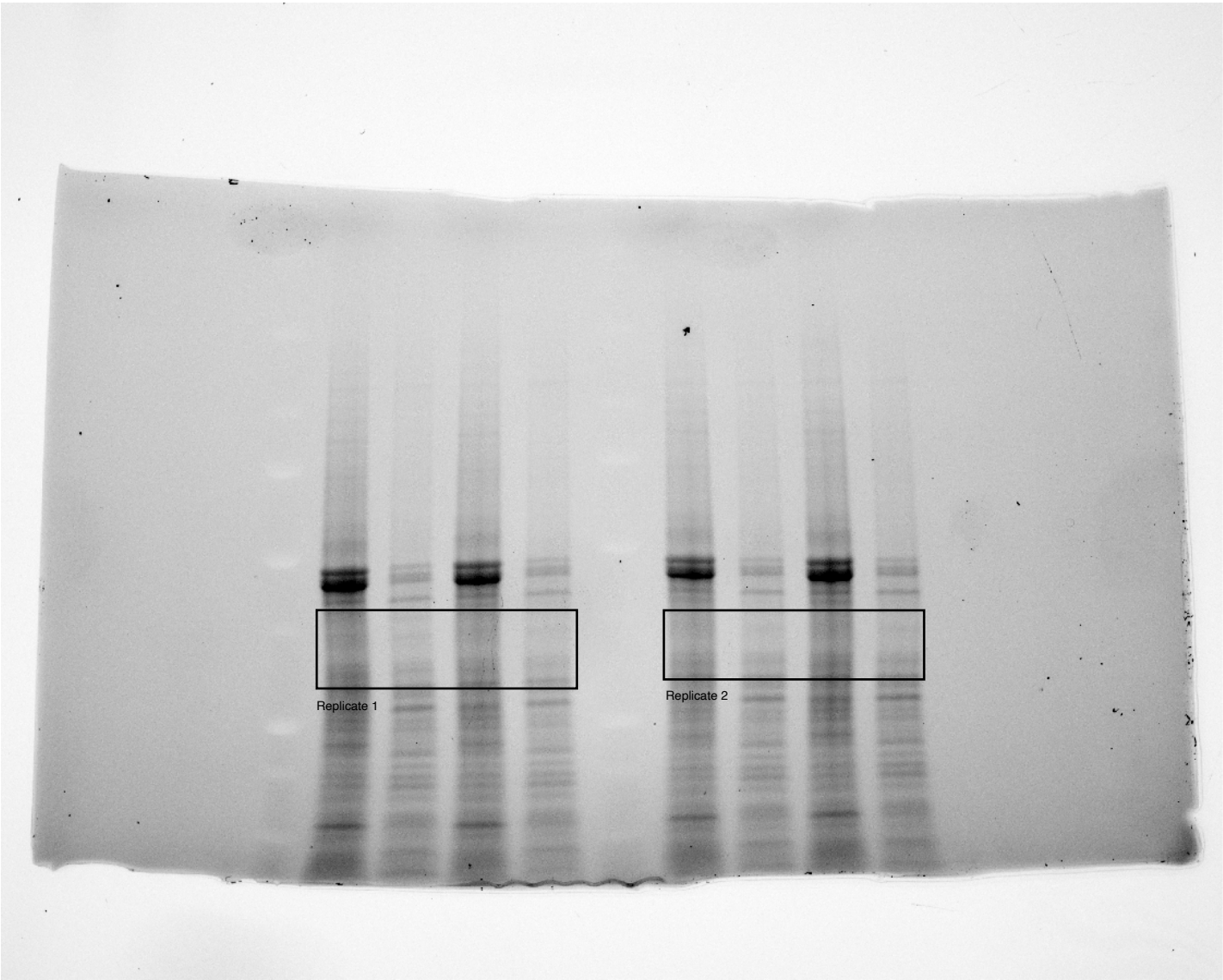

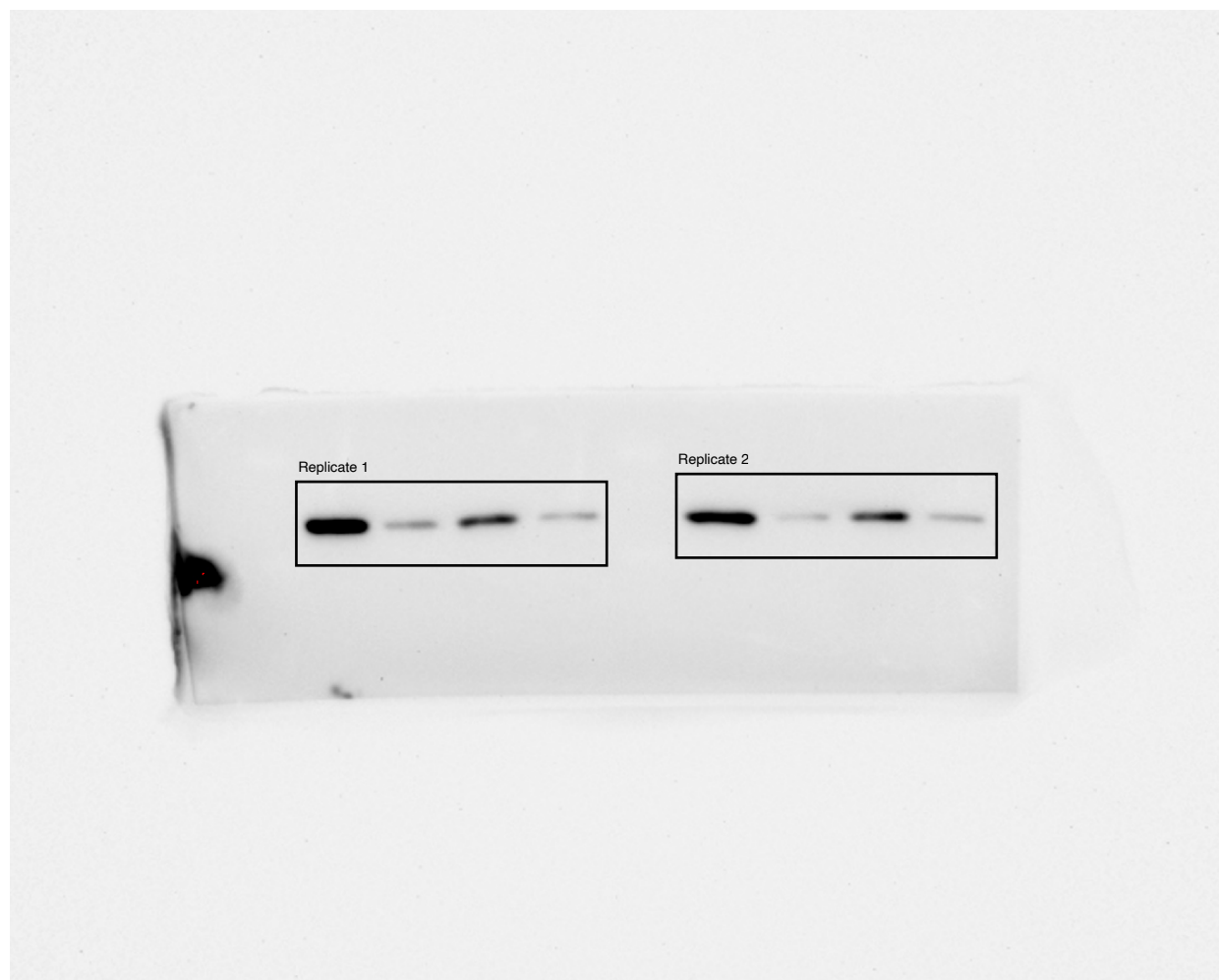

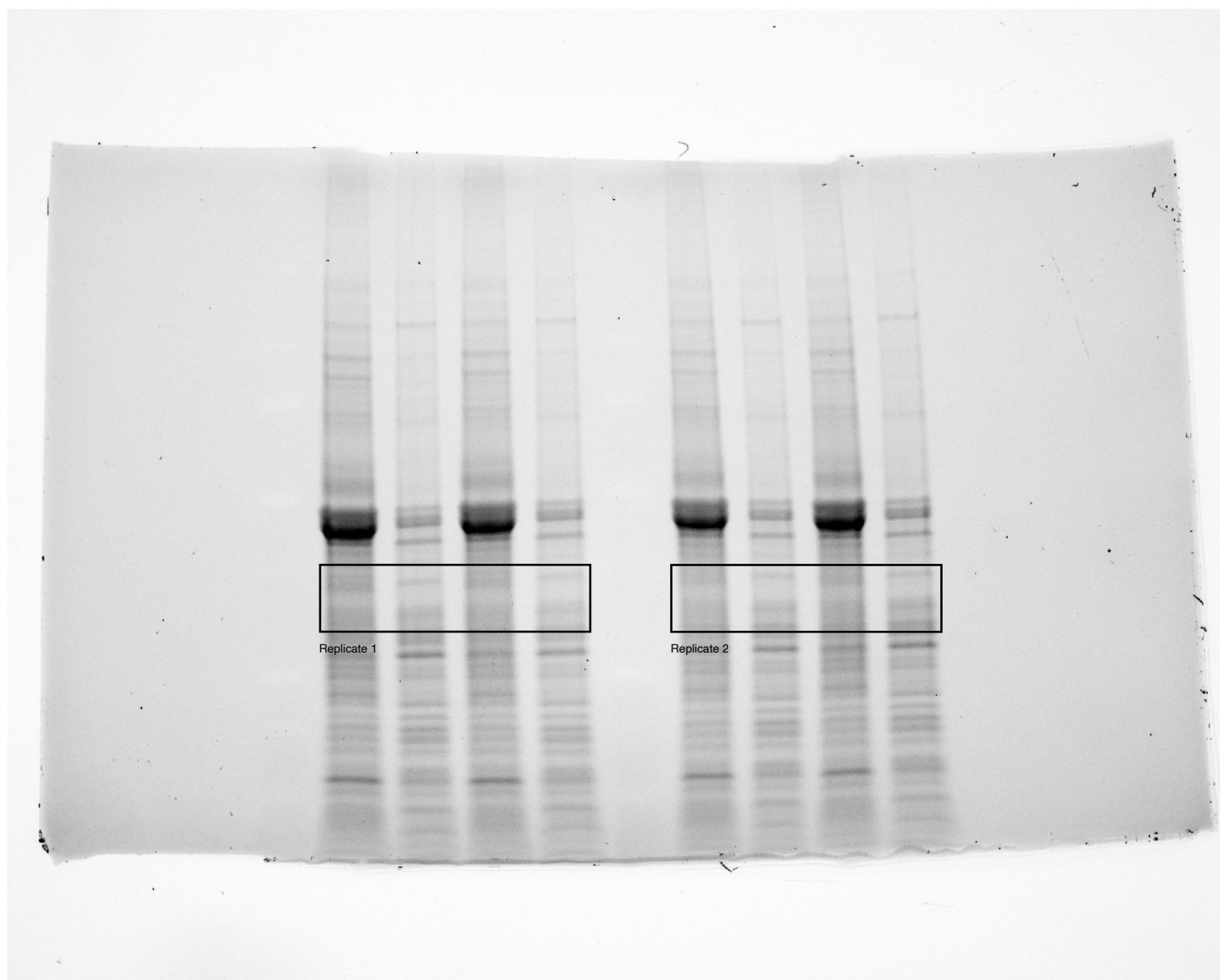

Supplement: SourceData F5 — is the source file for Fig. 5. [file jcb_202511182_sourcedataf5.pdf]

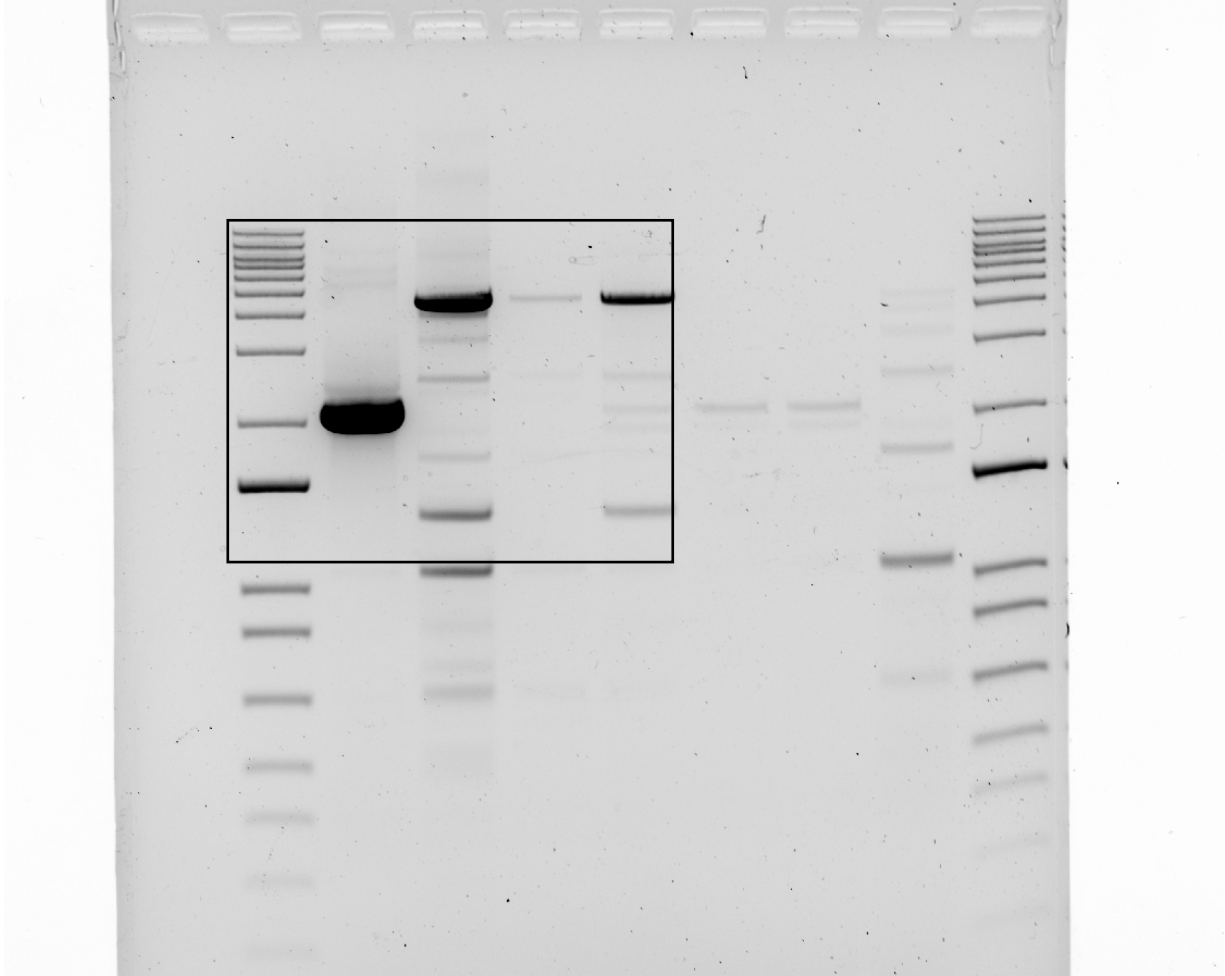

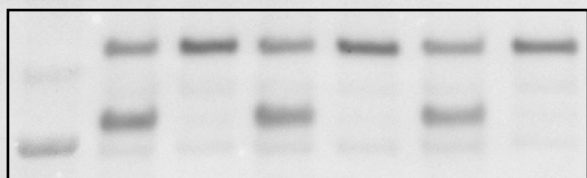

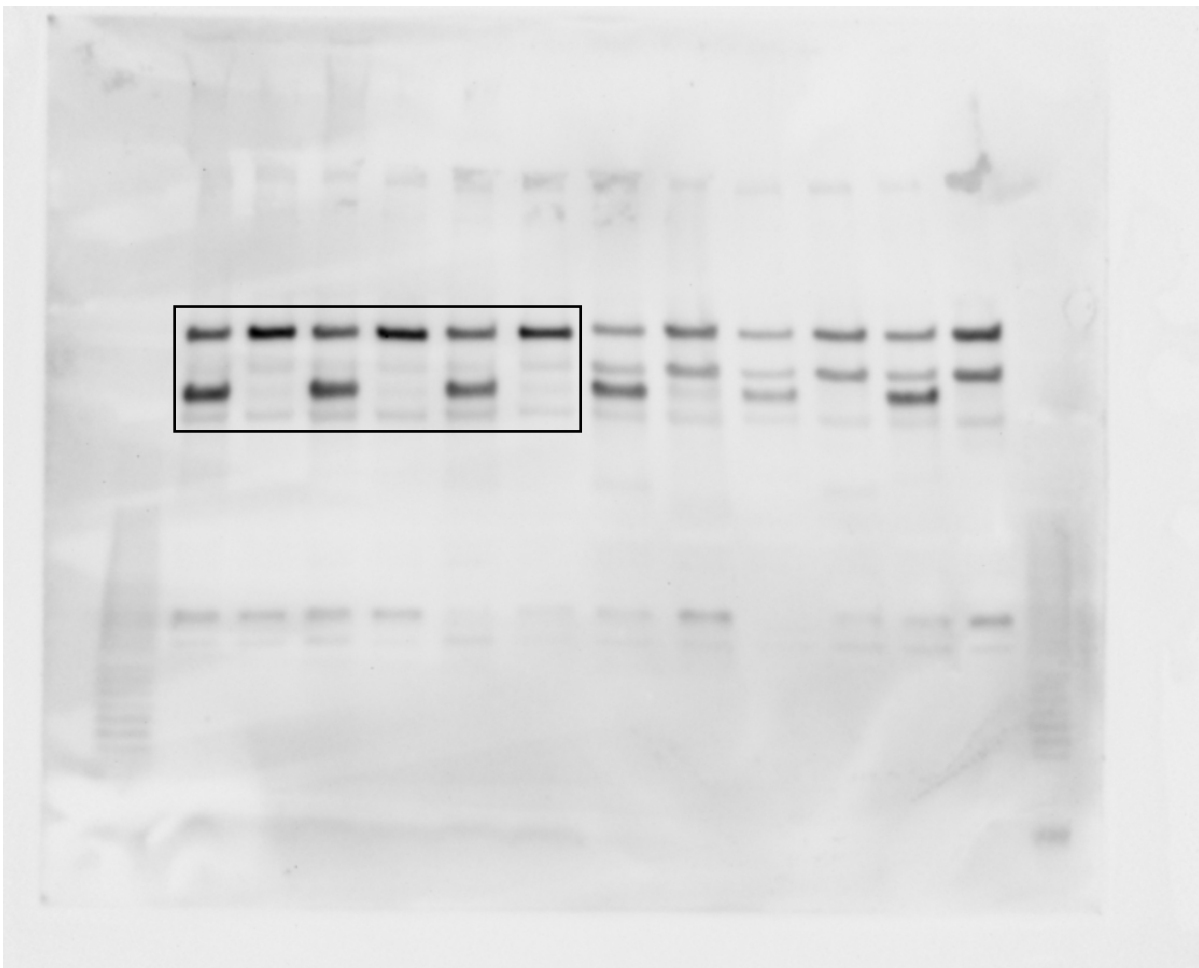

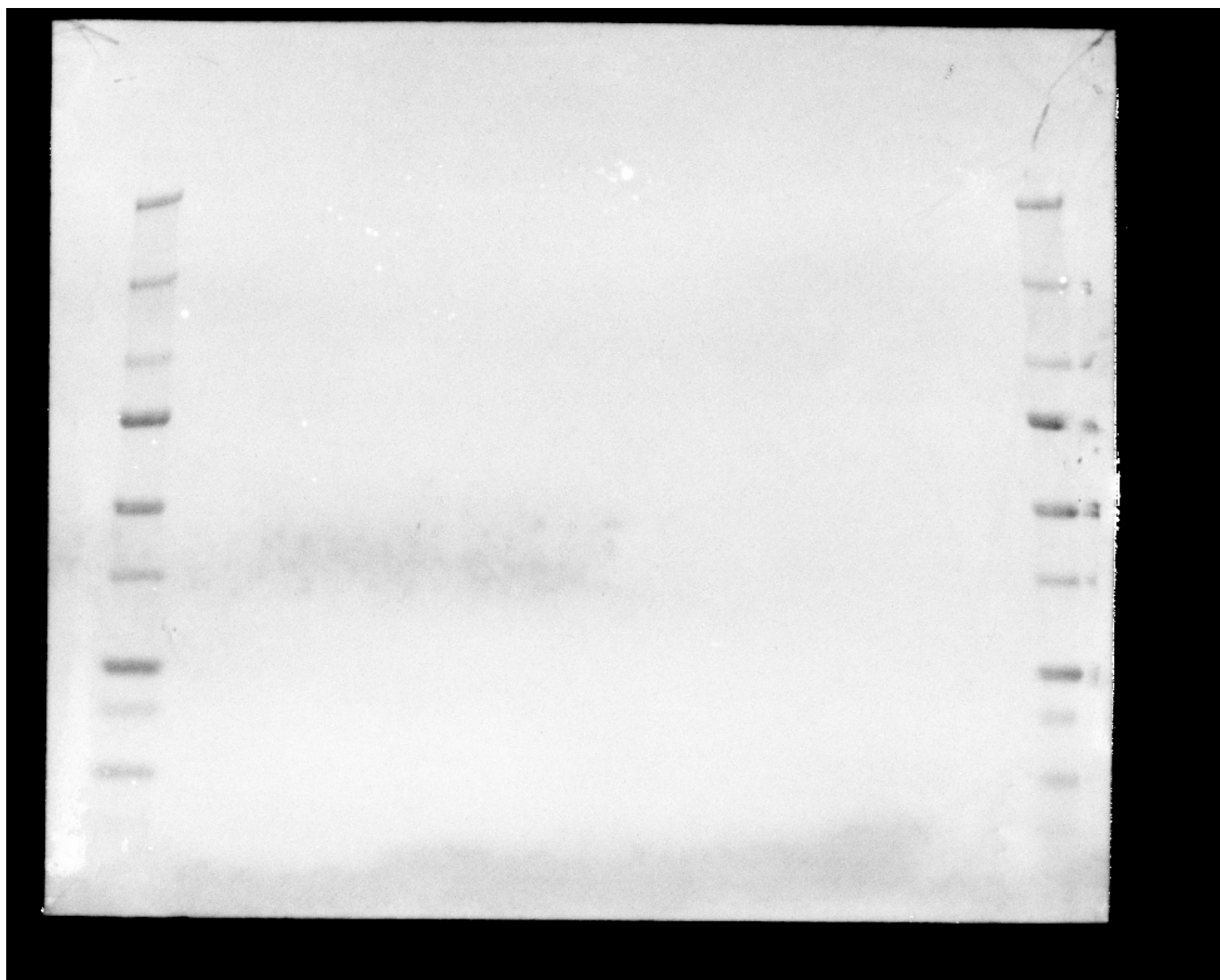

Supplement: SourceData FS2 — is the source file for Fig. S2. [file jcb_202511182_sourcedatafs2.pdf]

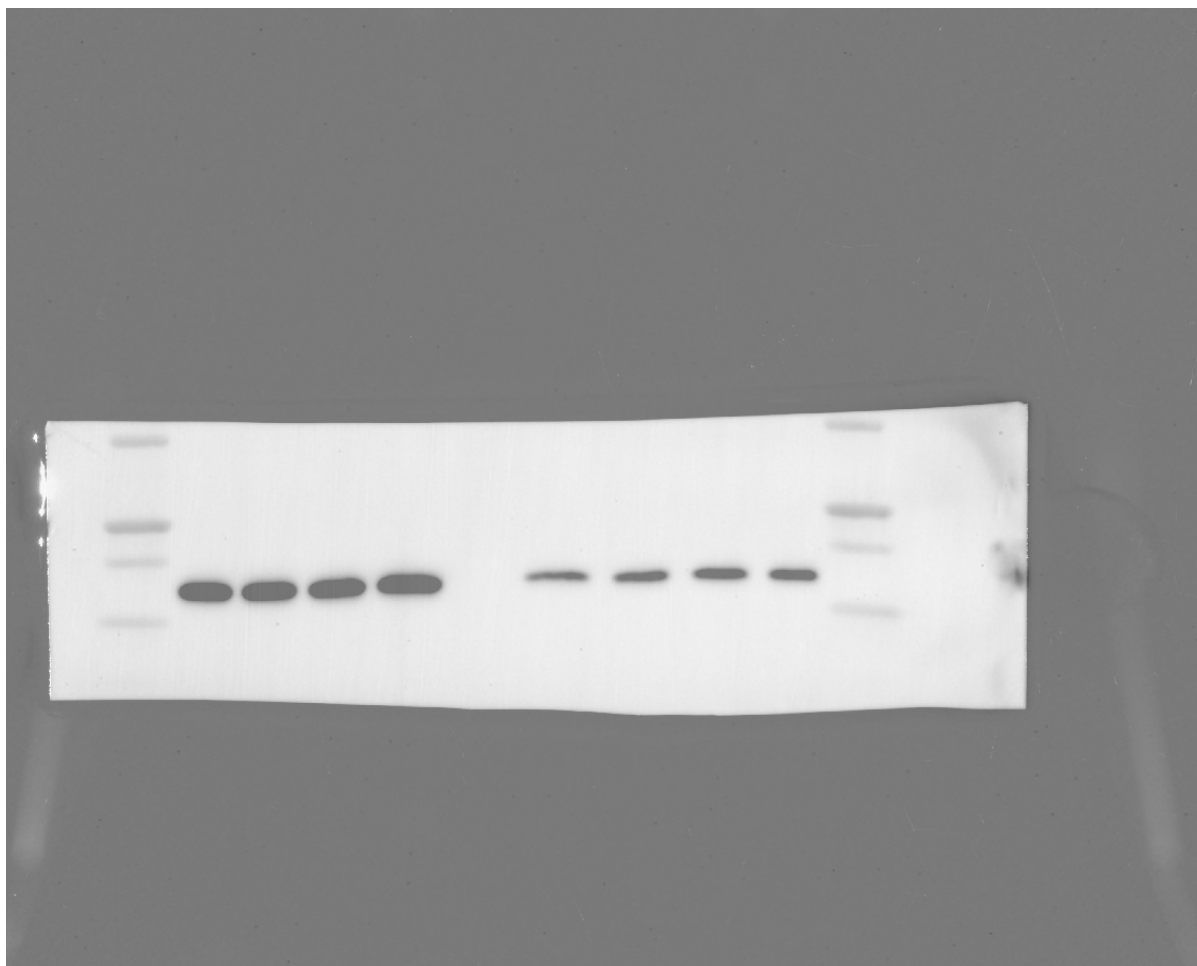

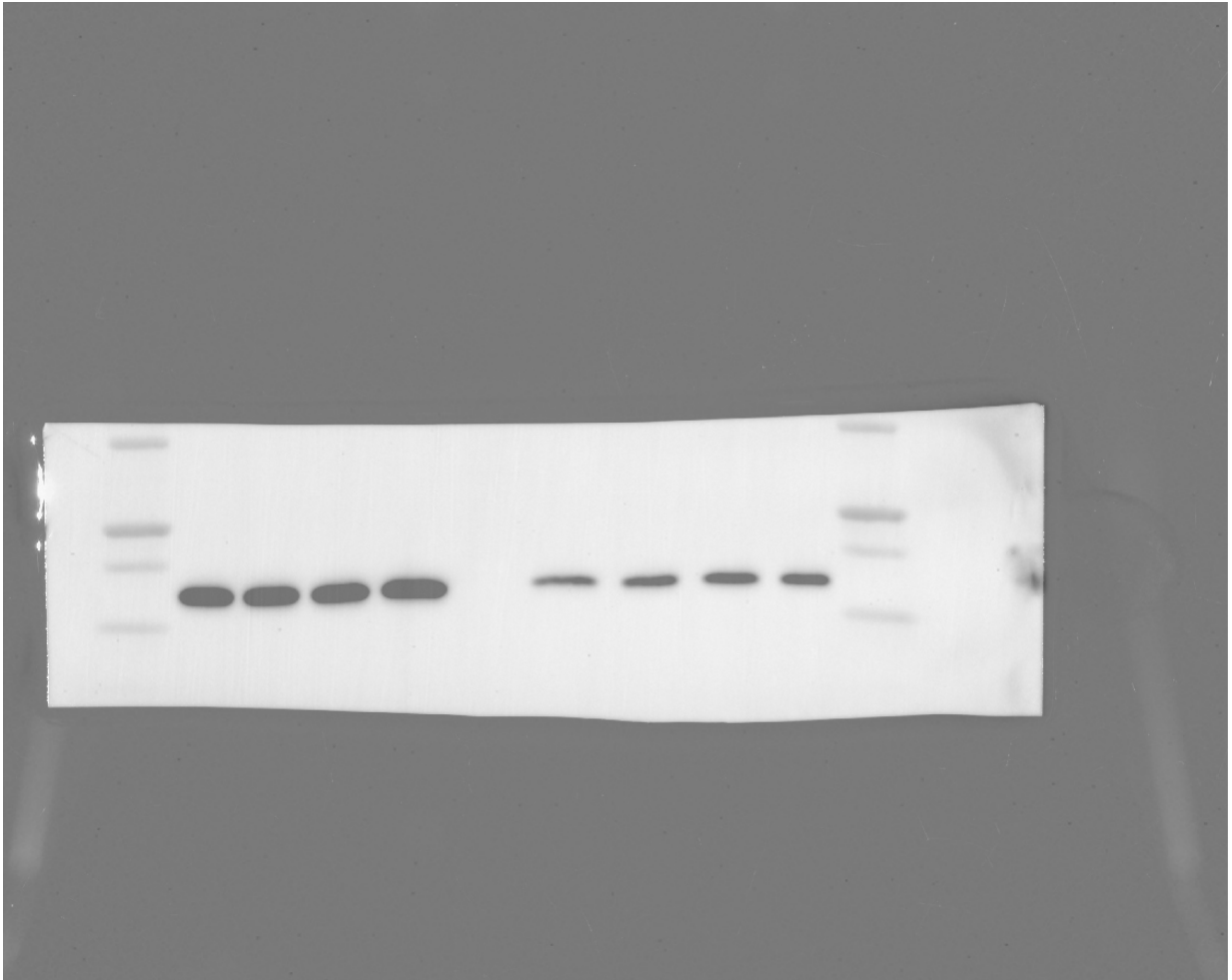

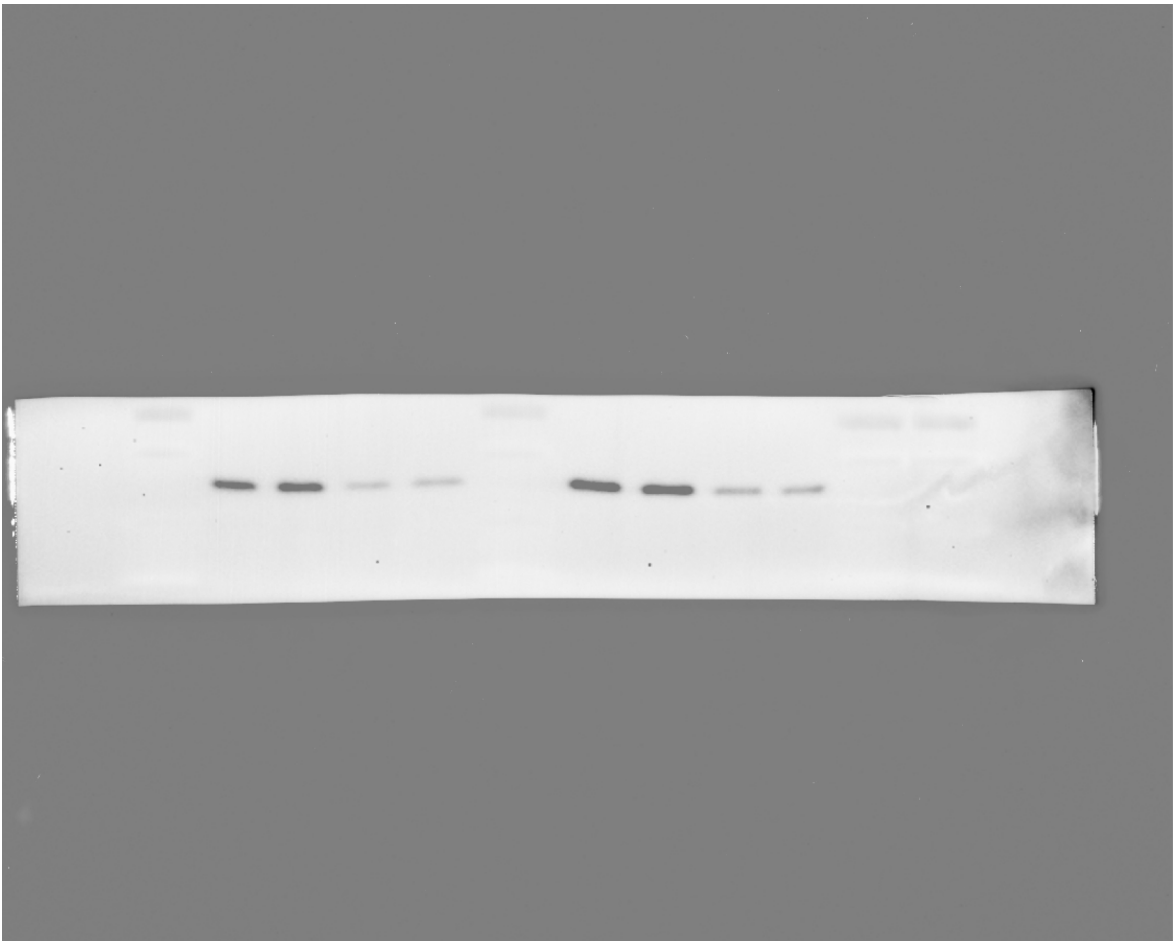

Supplement: SourceData FS3 — is the source file for Fig. S3. [file jcb_202511182_sourcedatafs3.pdf]

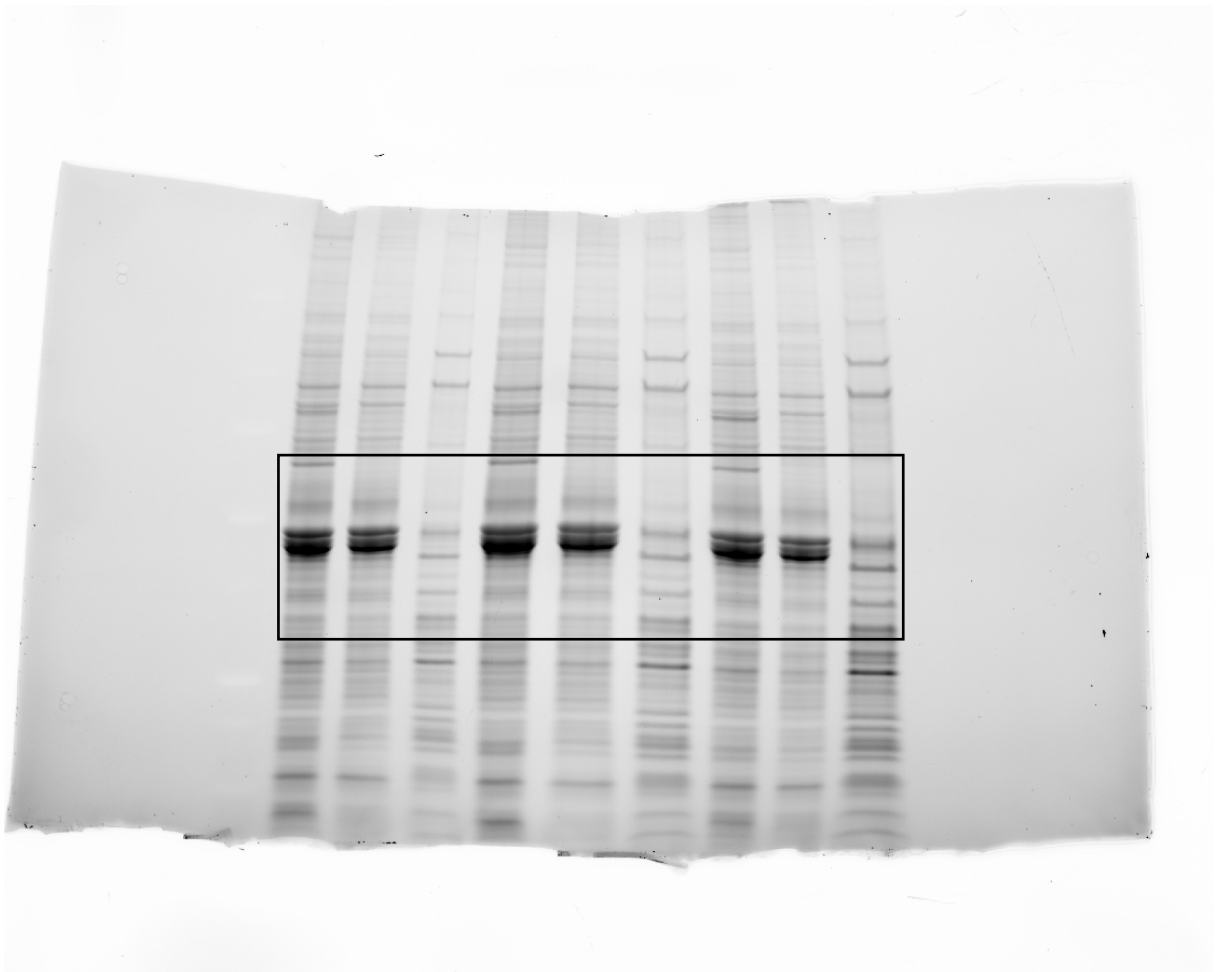

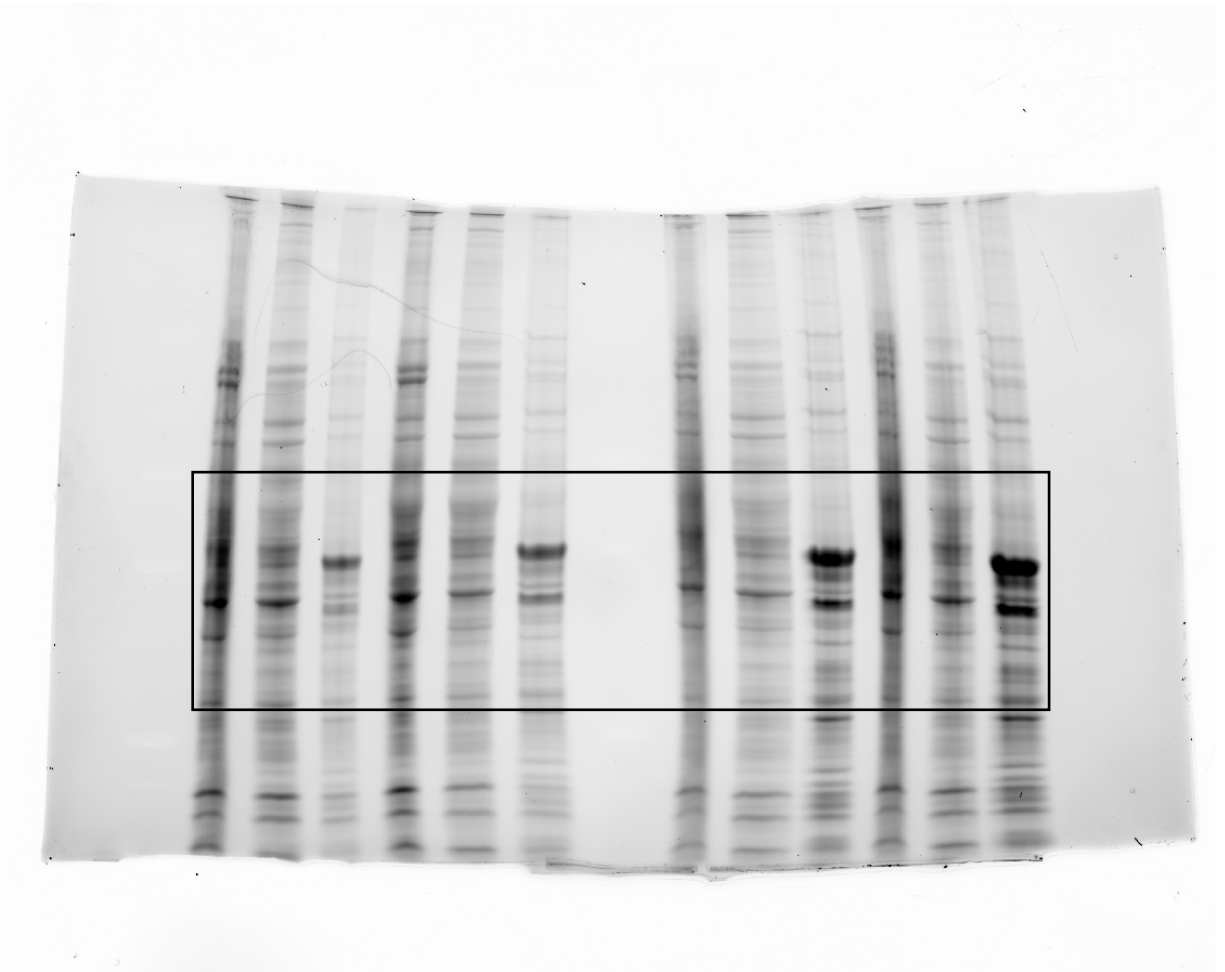

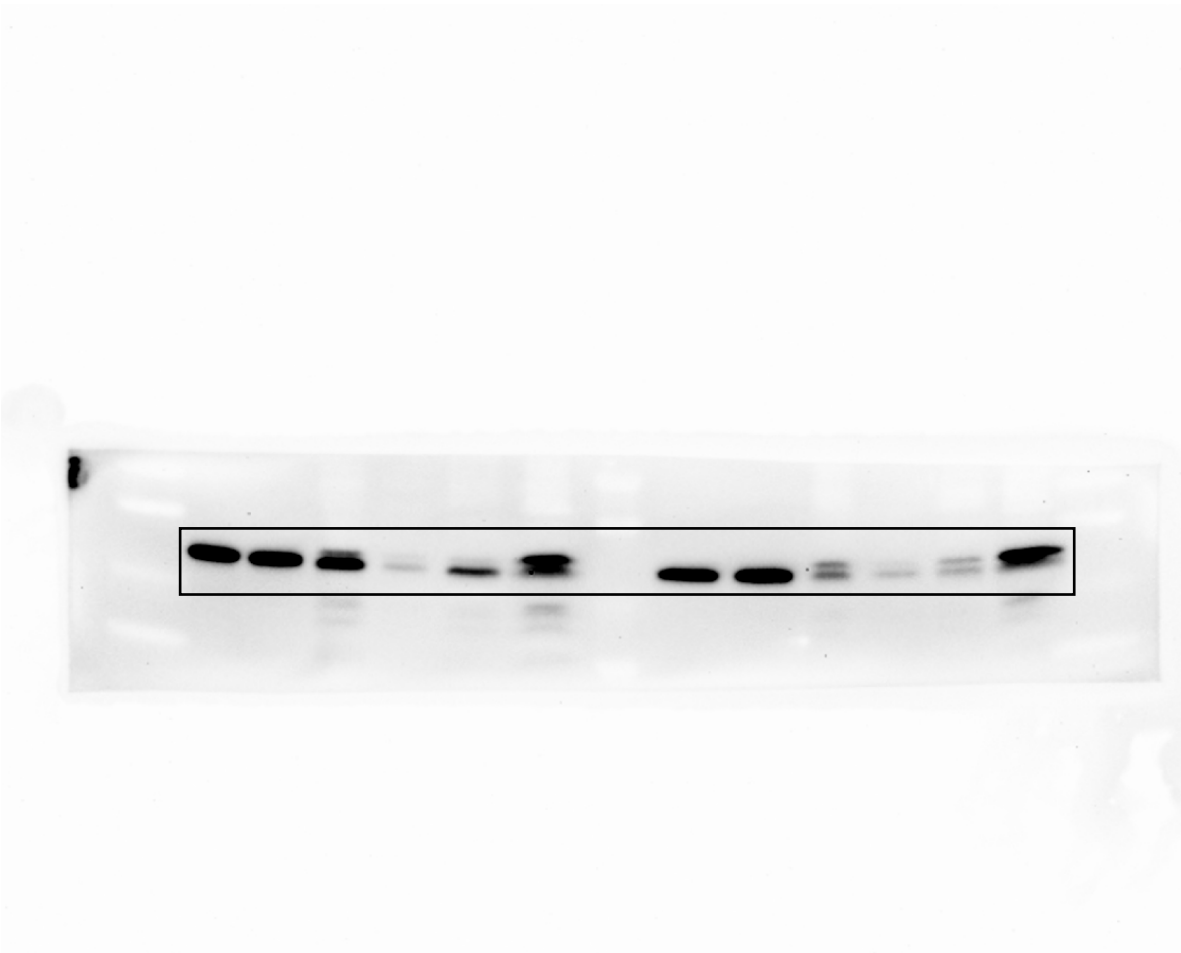

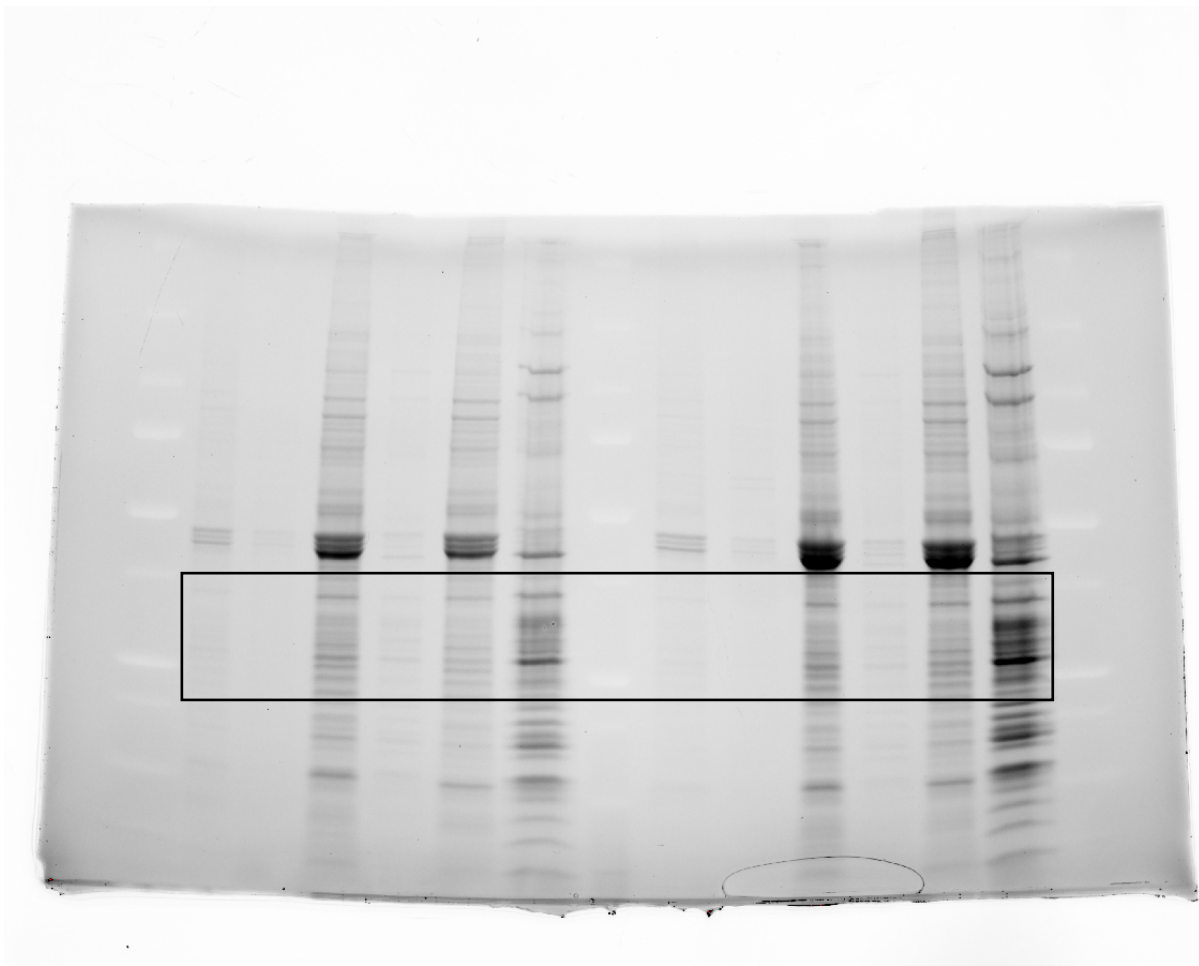

Supplement: SourceData FS4 — is the source file for Fig. S4. [file jcb_202511182_sourcedatafs4.pdf]

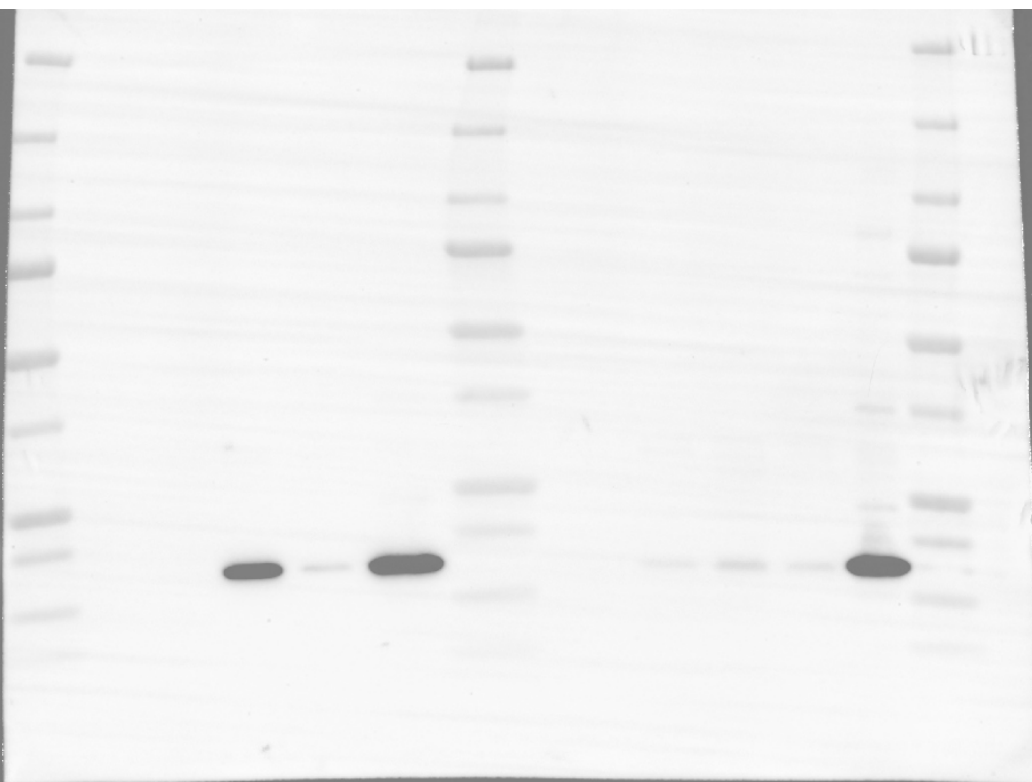

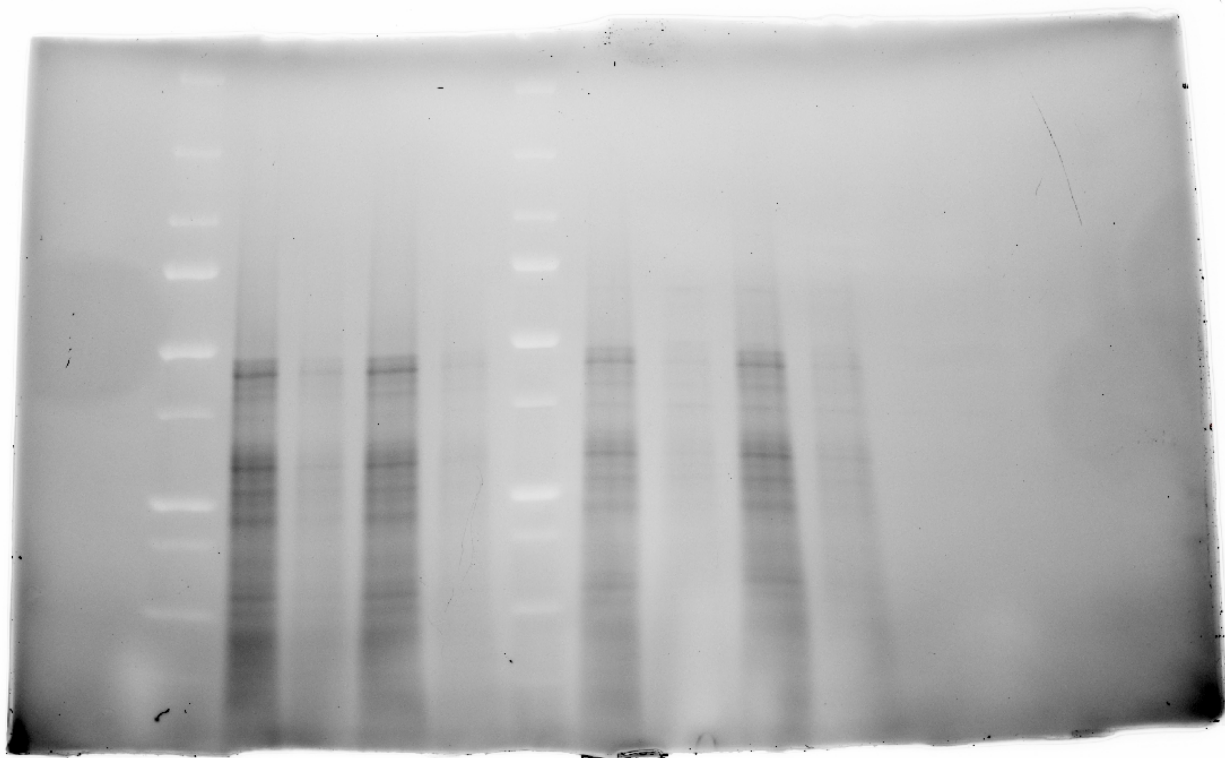

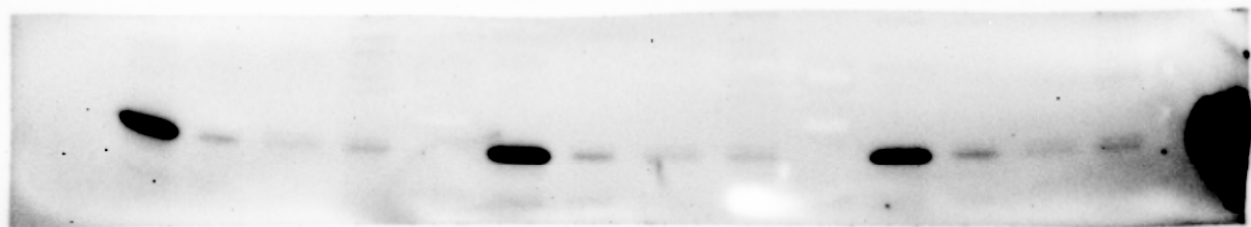

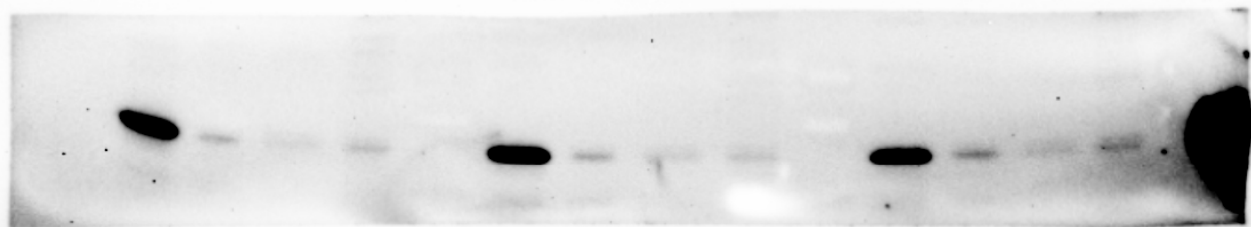

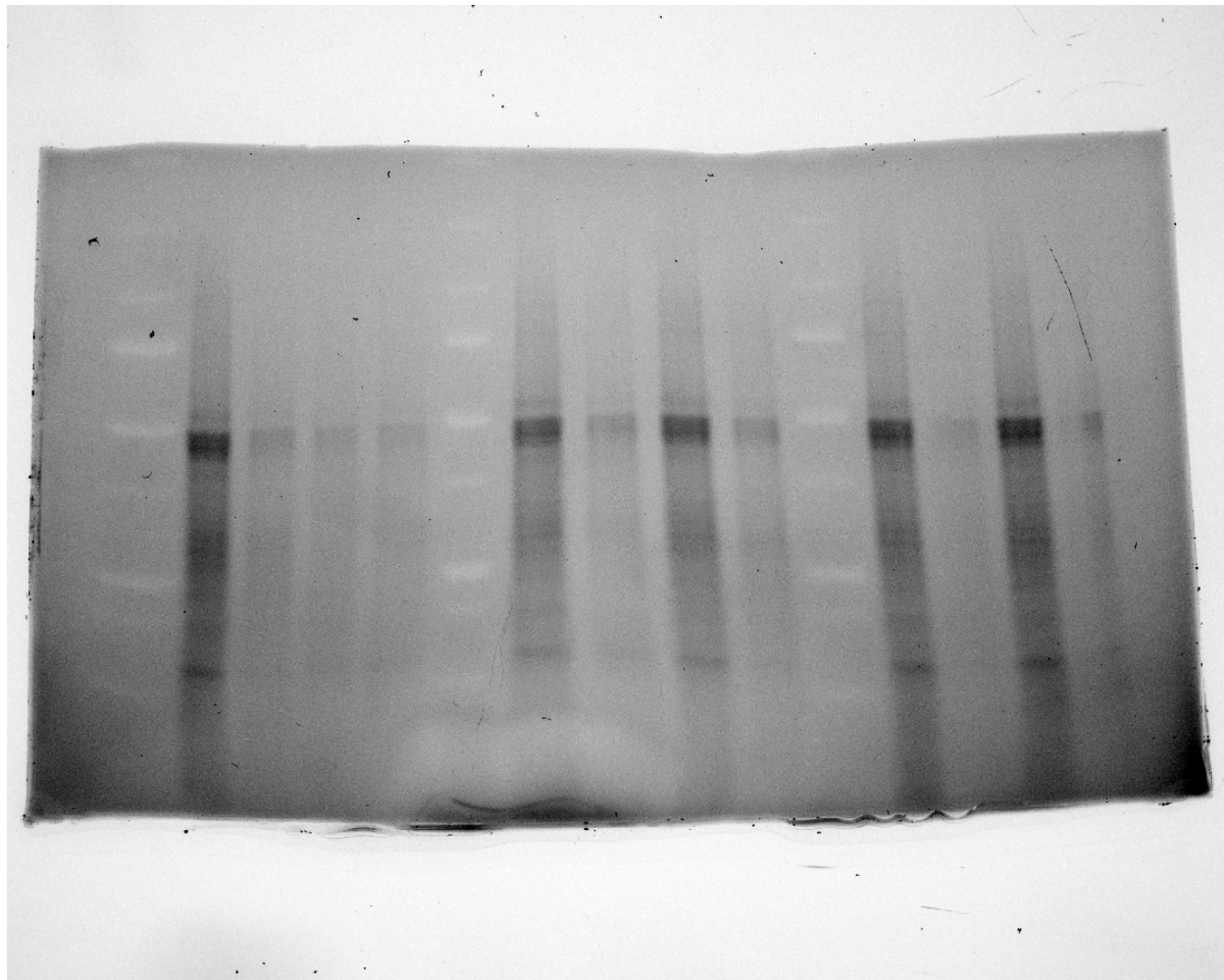

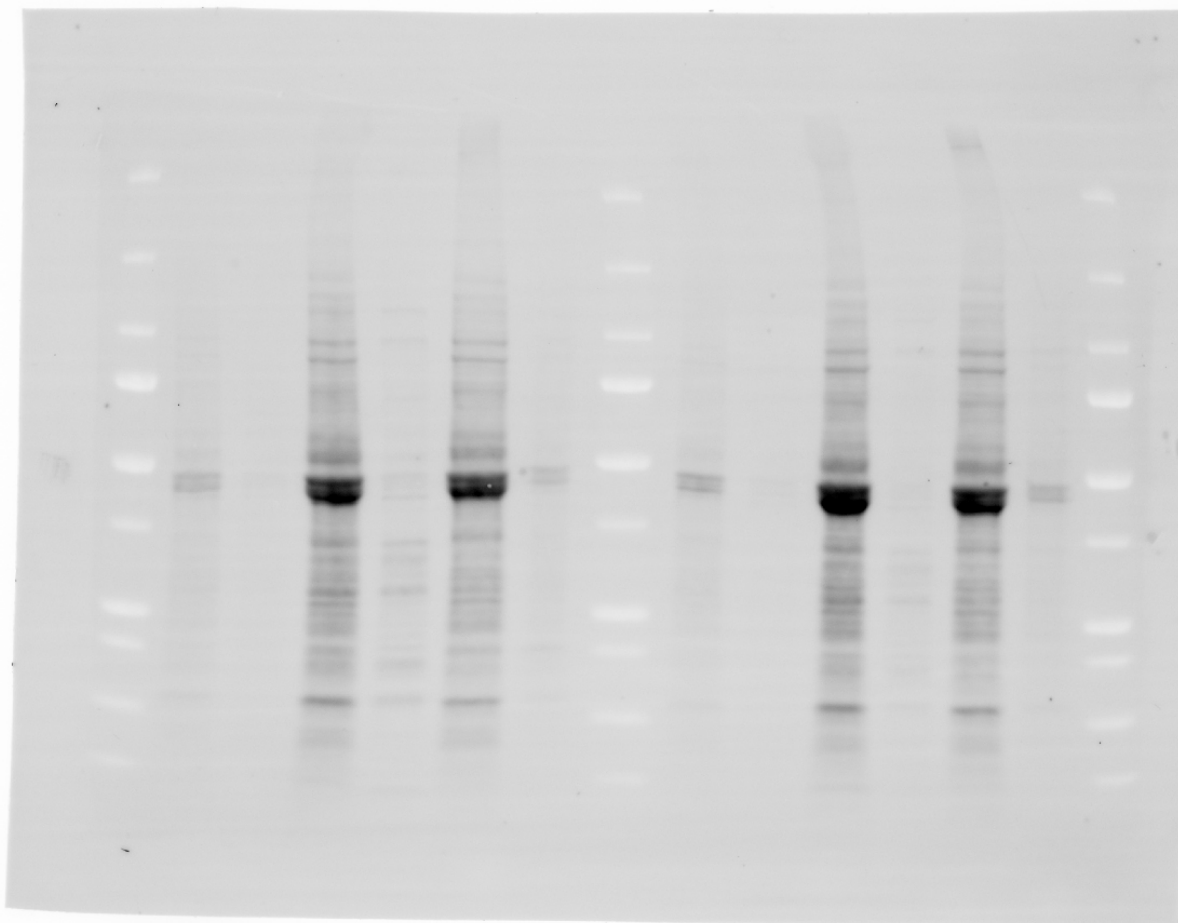

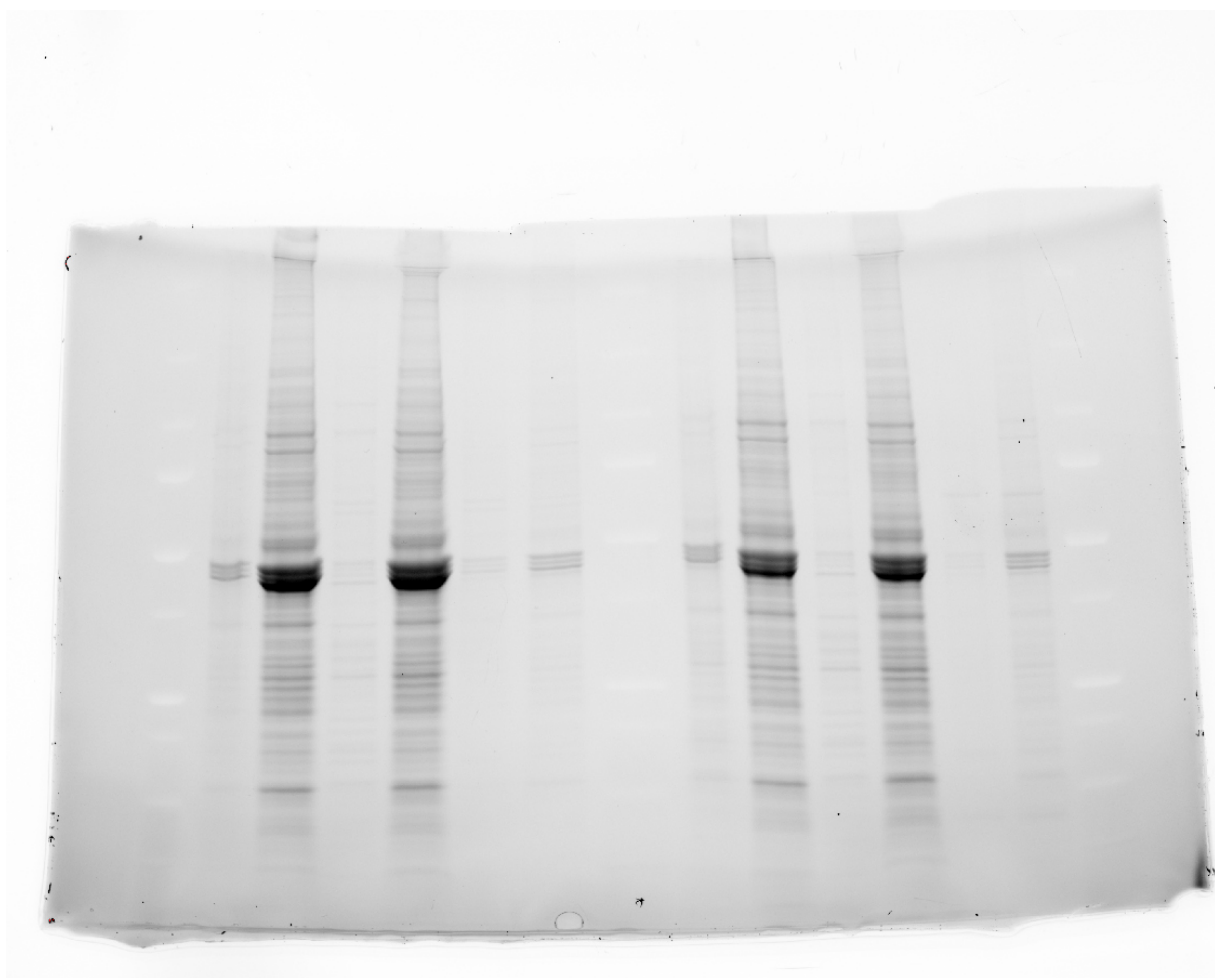

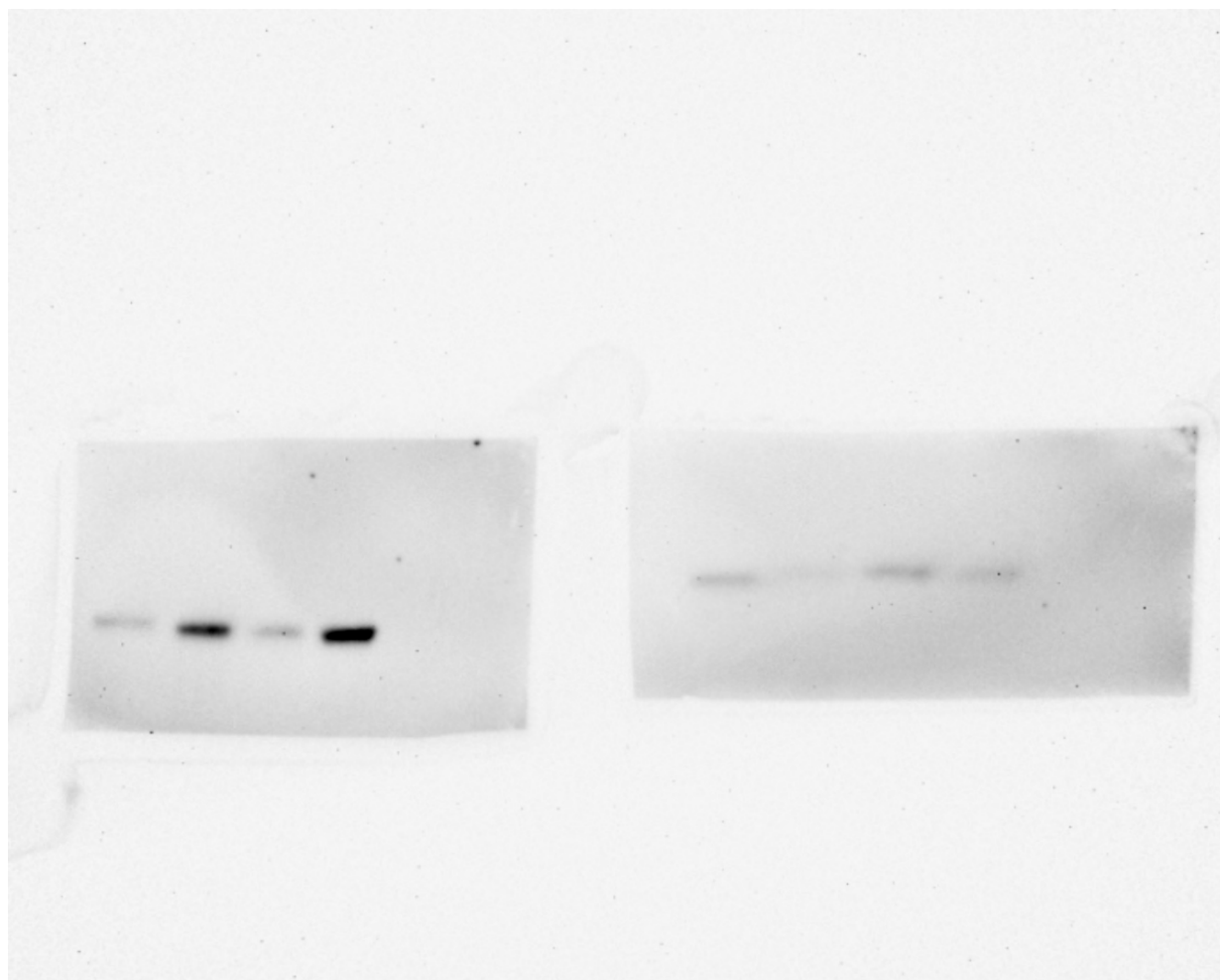

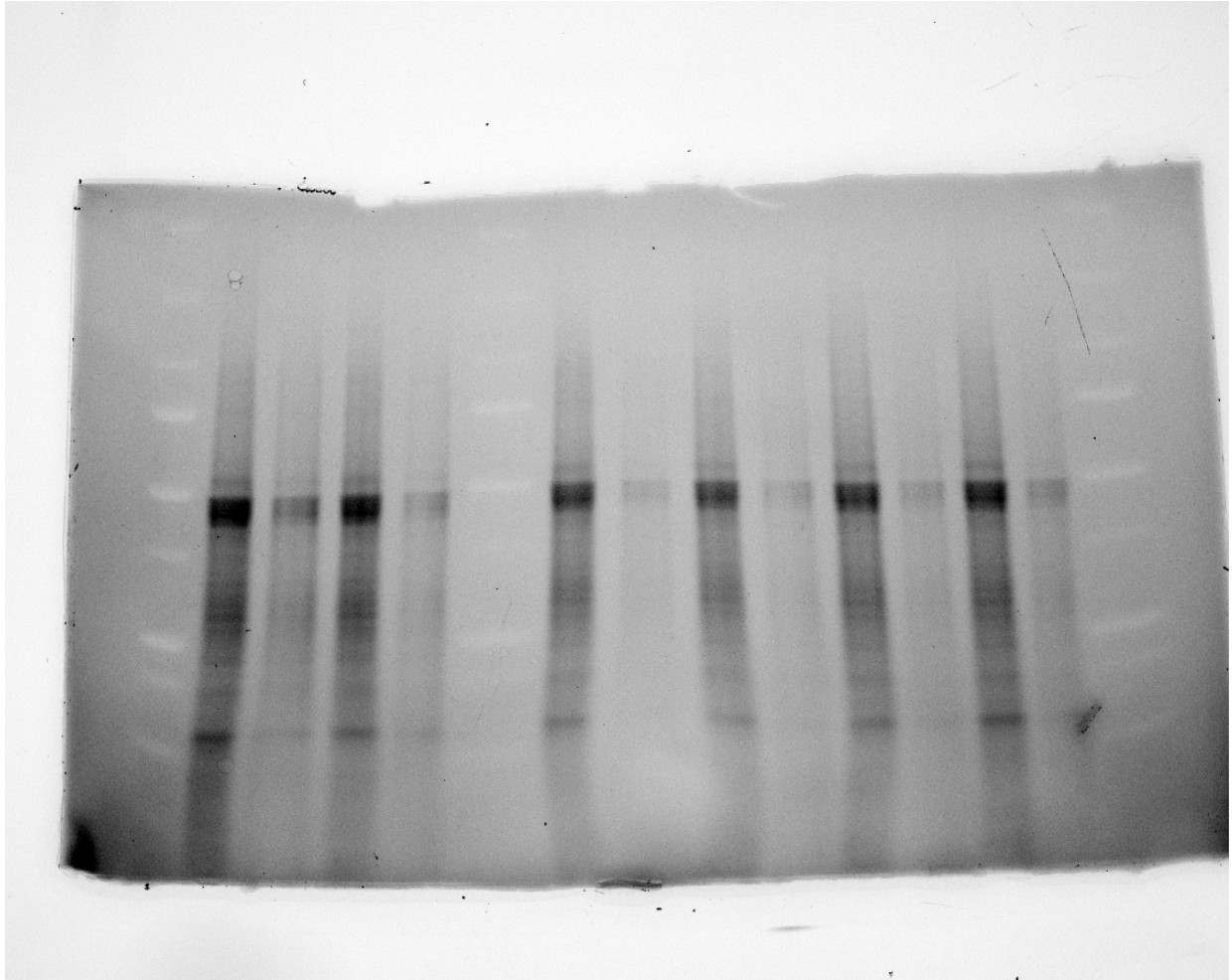

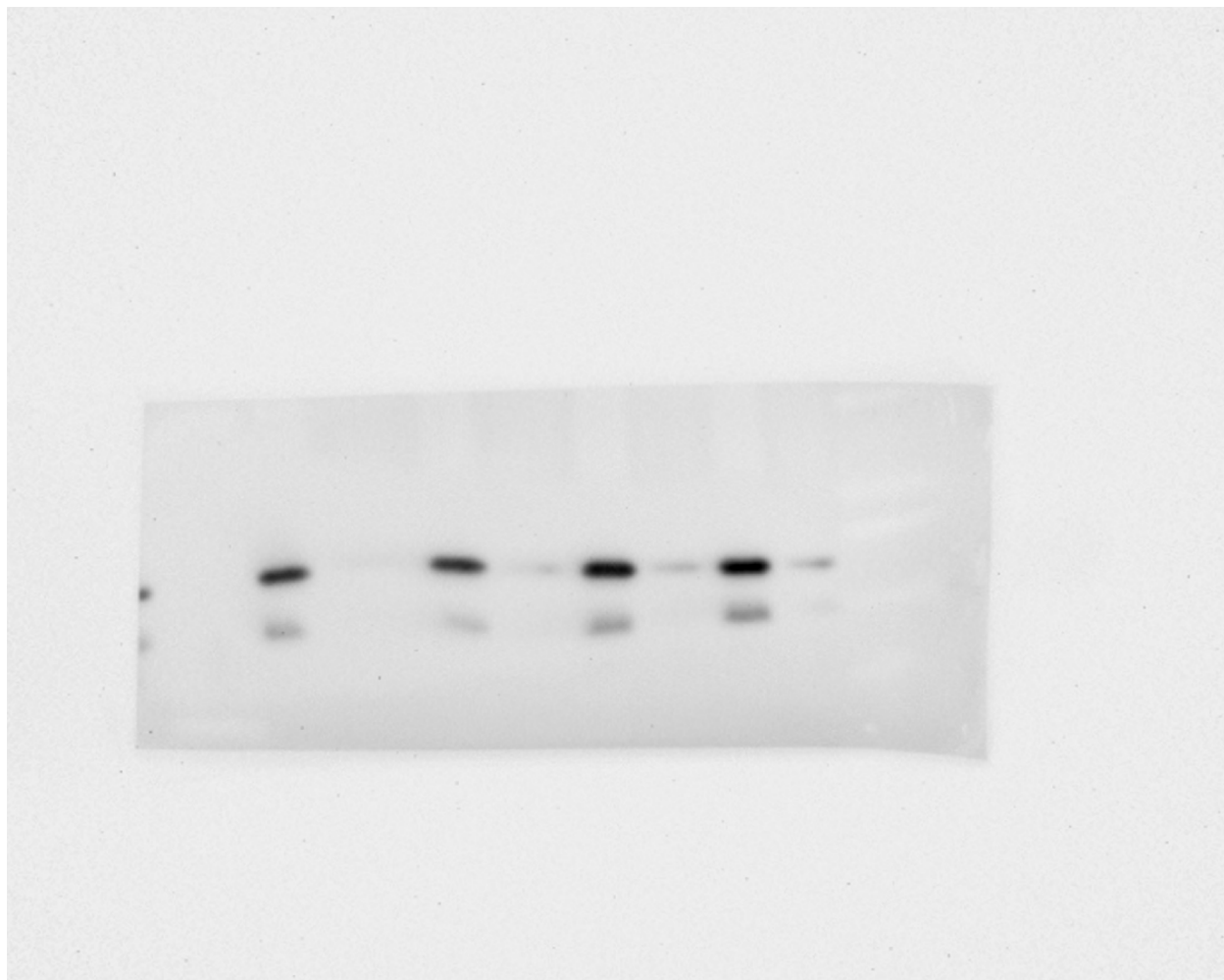

Supplement: SourceData FS5 — is the source file for Fig. S5. [file jcb_202511182_sourcedatafs5.pdf]
